# Melleins—Intriguing Natural Compounds

**DOI:** 10.3390/biom10050772

**Published:** 2020-05-15

**Authors:** Pierluigi Reveglia, Marco Masi, Antonio Evidente

**Affiliations:** 1 Dipartimento di Scienze Chimiche, Università di Napoli Federico II, Complesso Universitario Monte S. Angelo, Via Cintia 4, 80126 Napoli, Italy; pierluigi.reveglia@gmail.com (P.R.); marco.masi@unina.it (M.M.); 2 Dipartimento di Medicina Clinica e Sperimentale, Università di Foggia, Plesso di Medicina Viale Luigi Pinto 1, 71122 Foggia, Italy

**Keywords:** 3,4-dihydroisocoumarins, melleins, chemical characterization, biological activities, biosynthesis

## Abstract

Melleins are 3,4-dihydroisocoumarins mainly produced by fungi, but also by plants, insects and bacteria. These specialized metabolites play important roles in the life cycles of the producers and they are involved in many biochemical and ecological processes. This review outlines the isolation and chemical and biological characterizations of natural-occurring melleins from the first report of (*R*)-mellein in 1933 to the most recent advances in their characterization in 2019. In addition, the pathways that could be involved in mellein biosynthesis are discussed, along with the enzymes and genes involved.

## 1. Introduction

**Figure 1:**
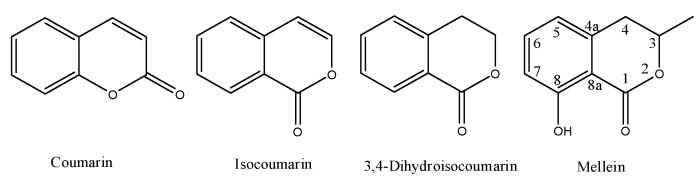
Structure of coumarin, isocoumarin, 3,4-dihydroisocoumarin and mellein.

Melleins are a subgroup of 3,4-dihydroisocoumarins. In general, these secondary metabolites belong to the class of polyketides. The 3,4-dihydroisocoumarins are also a subgroup of the isocoumarins, a well-known polyketides family that is the structural isomer of coumarin. The general moieties of these four groups of natural occurring compounds are reported in Figure 1, and their IUPAC names are chromen-2-one, 1*H*-isochromen-1-one, isochroman-1-one and 8-hydroxy-3-methylisochroman-1-one, respectively. 

The first coumarin was obtained as natural compound from *Coumarouna odorata* (tonka tree) [1], which is a species of flowering tree in the pea family (Fabaceae). The first report on 3,4-dihydroisocoumarin was in 1916 when the hydrangenol was isolated from *Hydrangea hortensia*, a species of flowering plants native to Asia and the Americas [2]. However, mellein is the best known in this subgroup, although previously it was named ocracin when isolated from the fungus *Aspergillus melleus* on 1933 [3]. Coumarin and isocoumarin derivatives are produced by bacteria, fungi, higher plants, insects, lichens, liverworts, and marine sponges. They showed different biological activities, such as antimicrobial, antitumor, antileukemic, and antiviral ones. Furthermore, they also exhibited toxicity as ochratoxin A, which is a mycotoxin, biosynthesized by *Aspergillus* and *Penicillium* species*,* which usually contaminates a variety of food imparting heavy toxicity against animals and humans [4]. The isolation of several coumarins, isocoumarins and 3,4-dihydroisocoumarins from different natural sources, and their important biological activities were covered by some previous reviews [5,6,7]. Some other reviews extensively describe the reaction sequences applied over the year for their synthesis [8,9,10].

The present review is focused on the intriguing melleins, a subgroup of isocoumarins; we report their natural sources, isolations and chemical and biological characterizations from their first isolation in 1933 till 2019. In addition, the biosynthetic pathways and genes involved in melleins production are also discussed.

## 2. Natural Sources, Isolation, Chemical Characterization and Biological Activities

### 2.1. Melleins from Fungi

**Figure 2:**
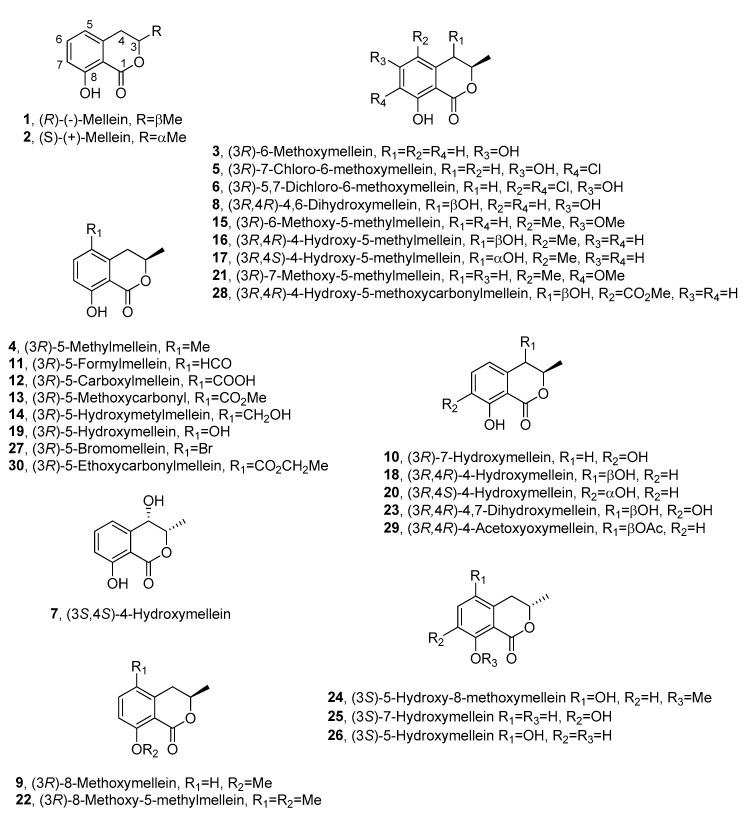
The structures of **1**–**30**.

Fungi are the most important source of melleins, and (*R*)-(-)-mellein (**1**, Figure 2) is the most common among this group. 

Compound **1** was isolated for the first time in 1933 from the fungus *Aspergillus melleus* [3] and named ocracin, as cited above. However, its structure was determined only in 1955 [11] and the *R* absolute configuration (AC) at C-3 was successively assigned [12,13]. Its enantiomer, the (*S*)-(+)-mellein (**2**, Figure 2), is also known as a natural product, but it is produced by few species of fungi compared with **1**. In particular, compound **2** was firstly isolated from an unidentified fungus [14] and then from the cultures of the insect pathogen *Fusarium larvarum,* together with five other secondary metabolites [15]. 

A structural analogue of **1** and **2** was isolated from *Sporormia bipartis* and characterized as 6-methoxymellein (**3**, Figure 2) [16]; another analogue was isolated from the plant pathogen *Fusicoccum amygdali,* together with the main phytotoxic metabolite fusicoccin A. The compound, whose structure was elucidated by NMR, was found to be 5-methylmellein (**4**, Figure 2). In this study, **4** inhibited conidia germination in some fungi, but it had no detectable phytotoxicity in vitro [17]. Compound **3** was obtained as the main metabolite produced by the fungus *Sporormia affinis* together with two chlorinated analogues, which were characterized as 7-chloro-6-methoxymellein (**5**, Figure 2) and 5,7-dichloro-6-methoxymellein (**6**, Figure 2) by NMR and mass spectra [18]. 

**Figure 3:**
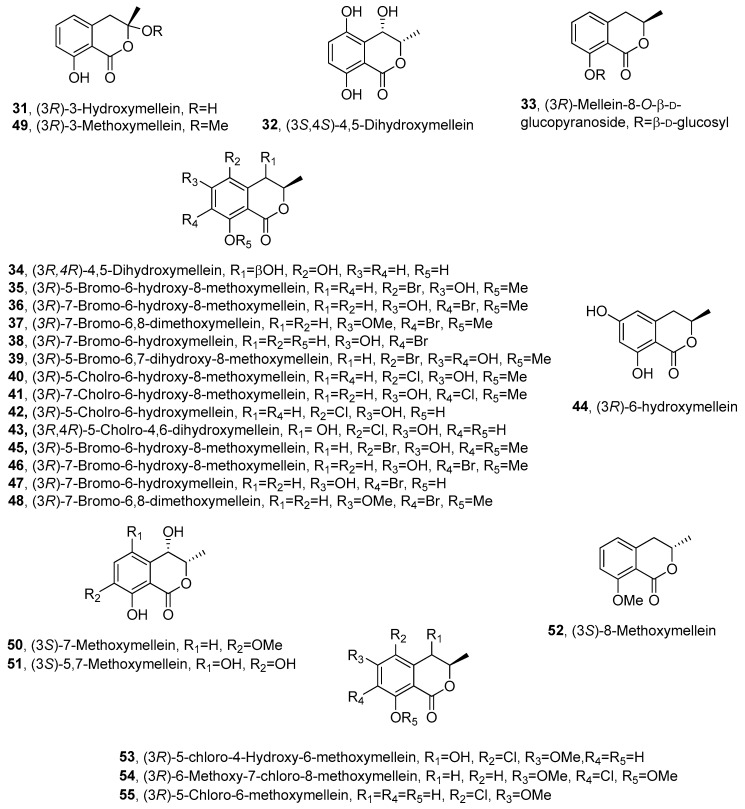
The structures of compounds **31**–**55**.

Studies carried out to evaluate the production of mycotoxins by *Aspergillus oniki* 1784, allowed to isolate **1** together with two related compounds, which were characterized as 3-methyl-4,8-dihydroxy-3,4-dihydroisocoumarin and 3-methyl-3,8-dihydroxy-3,4-dihydroisocoumarin. Their LD_50_ values for mice were measured by intraperitoneal injection, but their stereochemistry was not determined [19]. Successively, compound **1** together with *cis**-***4-hydroxymellein and other five metabolites were isolated from *Lasidiplodia theobromae* culture filtrates, which inhibited the green plant growth. However, for **1** and its analogues, no biological activity was reported [20]. One of the stereoisomers of 4-hydroxymellein was also isolated together with **1** from *Aspergillus ochraceus* [21,22]. The AC of one of the two enantiomers of *cis**-***4-hydroxymellein was determined when it was purified from the mycelium of *Cercospora taiwanensis,* together with **2**, and characterized as *cis*-(3*S*,4*S*)-4-hydroxymellein (**7**, Figure 2) [23]. Furthermore, a 4,6-dihydroxymellein (**8**, Figure 2) was isolated in a screening of 61 other species of *Cercospora* [24]. 

(*R*)-(-)-mellein (**1**), (*R*)-8-methoxymellein (**9**, Figure 2) and (3*R*)-7-hydroxymellein (**10**, Figure 2) were isolated from *Septoria nodorum* by Devys and co-authors and characterized by NMR spectroscopy [25].

A series of dihydroisocoumarin derivatives were isolated in a systematic study on metabolites of the Xylariaceous fungi belonging to *Hypoxylon* and *Numularia* species. 5-methylmellein (**4**) was produced by almost all species studied, whereas **1** was produced by *Hypoxylon fragiforme, Hypoxylon howeianum, Hypoxylon haematostroma, Hypoxylon venusfuissimum* and *Hypoxylon deustum.* In the same study, five other analogues of mellein were isolated and identified as 5-formyl- (**11**, Figure 2), 5-carboxy- (**12**, Figure 2), 5-methoxycarbonyl (**13**, Figure 2), 5-hydroxymethyl (**14**, Figure 2), and 6-methoxy-5-methyl- (**15**, Figure 2) mellein. **11** was produced by *Numularia discreta* and *Numularia broomiana,* **12** by *Hypoxylon mammatum, Hypoxylon illitum* and *N. discreta,* **13** by *H. mammatum,* **14** by *H. illitum* and **15** by *Hypoxylon atropunctatum* [26].

Mellein 5-carboxylic acid (**12**), together with 5-methyl mellein (**4**), (*S*)-(+)-mellein (**2**) and other compounds, was isolated from *Phomopsis oblonga* in a study for the production of elm bark beetle boring and feeding deterrents. Compound **2** was obtained as minor metabolite, while **4** and **12** were active against adult females of *Scolytus* sp. beetles [27]. The AC at C-3 of (-)-5-carboxylmellein (**12**) and (-)-5-hydroxylmethylmellein (**15**) was assigned as *R* by chemical correlations when they were isolated from *Valsa ceratosperma*, a fungus inducing apple canker. The same fungus also synthesizes (-)-5-methylmellein (**4**), and two new compounds which were characterized as *cis*-(3*R*,4*R*)-(-)-4-hydroxy-5-methylmellein (**16**, Figure 2) and *trans-*(3*R*,4*S*)-(+)-4-hydroxy-5-methylmellein (**17**, Figure 2). All the five compounds were phytotoxic to apple shoots and lettuce seedlings [28]. 

The first report of **1** in the genus *Nectria* occurred in 1986 when it was isolated from *Nectria fuckeliana* by Ayer and Shewchuk [29]. 

Five phytotoxic metabolites were purified from the culture filtrates of *Botryosphearia obtusa* (Schw.) Shoemaker, the causal agent of frogeye leaf spot and fruit black rot of apple. Among them, (*R*)-(-)-mellein (**1**), *cis*-(3*R*,4*R*)-(-)-4-hydroxymellein (**18**, Figure 2) and (3*R*)-5-hydroxymellein (**19**, Figure 2) were identifiedusing spectroscopic and optical methods [30]. **19** was isolated for the first time as a fungal metabolite and the phytotoxicity of all metabolites was tested by leaf puncture assay on seventeen apple cultivars and eight weed species. The apple cultivars Supergold and Silverspure and the weeds species prickely sida and morning glory were the most sensitive species to all the phytotoxins [31]. Successively, (*R*)-(-)-mellein (**1**) was isolated as a phytotoxic metabolite, together with citrinin, tyrosol and α–acetylorcinol, from *Stagonospora apocynin*, which caused leaf spot disease on hemp dogbane (*Apocynum cannabinum* L.). All the compounds were non–specific toxins causing necrosis when assayed on hemp dogbane leaves, and those of eight other weed species [32]. 

In a study carried out to select in vitro wheat embryos with a high level of resistance, **1** was identified in the fungal organic extract of *Septoria nodorum* (Berk.) [33]. (3*R*,4*S*)-(-)-4-hydroxymellein (**20**, Figure 2), together with (3*R*,4*R*)-(-)-4-hydroxymellein (**18**), was isolated for the first time from the same organic extract [34]. *Phoma tracheiphila*, the fungus responsible for “mal secco” citrus disease, also produced (*R*)-(-)-mellein (**1**). Compound **1** was phytotoxic when tested at a concentration of 100 mg/mL in tomato cuttings and caused 100% mortality of *Artemia salina* larvae at 200 mg/mL [35]. 

Compound **1** was isolated together with other four compounds in a study conducted on 85 *Pezicula* strains isolated as endophytes from living branches of ten deciduous and coniferous trees. All the compounds demonstrated strong fungicidal and herbicidal activity, and to a lesser extent, algicidal and antibacterial activity. Their production was taxonomically significant [36]. 

(*R*)-(-)-mellein (**1**), (3*R*,4*R*)-hydroxymellein (**18**) and (3*R*,4*S*)-hydroxymellein were also isolated from *Microsphaeropsis* sp. in a study conducted on some fungi obtained from marine sponges. In the same study (3*R*)-6-methoxymellein (**3**) and (3*R*)-6-methoxy-7-chloromellein (**5**) were also isolated from the culture extracts of the *Coniothyrium* sp. All these mellein analogues showed antifungal activity against *Eurotium repens* and *Ustilago violacea* [37].

A crude extract of *Aspergillus ochraceus* inhibited the final stage of hepatitis C virus (HCV) replication. A bio-guided purification of the extract afforded the known (*R*)-(-)-mellein (**1**), together with circumdatins G and F, which were identified by NMR spectroscopy. Compound **1** inhibited HCV protease with an IC_50_ value of 35 μM [38]. 

Two novel melleins, namely, 7-methoxy-5-methylmellein (**21**, Figure 2) and 8-methoxy-5-methylmellein (**22**, Figure 2), together with three known dihydroisocoumarins and a tetralone derivative, were isolated from a culture filtrate of *Cytospora eucalypticola*. Their structures were elucidated by spectroscopic methods. Compounds **21** and **22** showed moderate antifungal and antibacterial activity against Gram-positive bacteria [39].

*Apiospora montagnei*, a marine fungus, isolated from the North Sea alga *Polysiphonia violacea,* produced a plethora of secondary metabolites comprising (*R*)-(-)-mellein (**1**) and (*R*)-8-methoxymellein (**9**) [40]. Two novel compounds, namely, the monomethyl ester of 9-hydroxyhexylitaconic acid and the (-)-enantiomer of the known (+)-hexylitaconic acid, were also isolated and characterized by spectroscopic methods. The crude fungal extract and all the metabolites did not show antibacterial, antifungal and antialgal activity [40].

*Sphaeropsis sapinea* was isolated from declining pine (*Pinus radiata*) plants in Sardinia and studied for its ability to produce phytotoxic metabolites [41]. *S. sapinea* was grown in liquid culture and the purification of the corresponding organic extract afforded the three already known (*R*)-(-)-mellein (**1**), (3*R*,4*R*)-4-hydroxymellein (**18**) and (3*R*,4*S*)-4-hydroxymellein (**20**) isolated for the first time from this fungus. When assayed for phytotoxic and antifungal activities on host and non-host plants and on some phytopathogenic fungi, **1** was the most active compound, while **18** and **20** showed only a synergic effect in both tests [41]. The same melleins were isolated from *Botryosphaeria mamane* PSU-M76, along with other three known secondary metabolites and a dihydrobenzofuran derivative named botryomaman [42]. Their antibacterial activity against *Staphylococcus* *aureus* ATCC 25923, *S. aureus* SK1 and compounds **1**, **18** and **20** was tested; they were inactive with equal MIC values of > 128 μg/mL [42].

Among a collection of 250 foliar endophytes of *Picea glauca,* several isolates produced toxic metabolites against *Choristoneura fumiferana* (spruce budworm) [43]. Three of them (strains CBS 120381, CBS 120379 and CBS 120380) were selected for isolation and characterization of phytotoxic metabolites by LC-MS and LC-NMR. The DNA sequencing revealed that CBS 120381 belongs to the Xylariaceae and it was near but not identical to *Nemania serpens*. The sequencing data indicated that CBS 120379 and CBS 120380 were both species of *Lophodermium*, with CBS 120379 most like fungi in the *Rhytistimataceae*. Two of them produced at least one mellein. CBS 120379 synthesized (*R*)-(-)-mellein (**1**), while CBS 120381 produced three known melleins: 5-methyl- (**4**), 5-formyl- (**11**) and 5-methoxycarbonyl- (**13**) mellein [43].

*Botryosphaeria obtusa*, the teleomorph of *Diplodia seriata*, is a pathogen associated with grapevine Botryosphaeria dieback. The fungus was studied for its ability to synthesize phytotoxic metabolites, which were purified and characterized by spectroscopic data [44]. The three known melleins, (*R*)-(-)-mellein (**1**), (3*R*)-7-hydroxymellein (**10**) and (3*R*,4*R*)-4-hydroxymellein (**18**), and a novel mellein characterized as 4,7-dihydroxymellein (**23**, Figure 2) were isolated from the liquid culture of this fungus [44]. When assayed on grapevine leaves, **23** was the most active compound, inducing full leaf necrosis with minimum inhibitory concentrations (MIC) of 2 μg/mL [44]. Other Botryosphaeriaceae, such as *Bothryosphaeria rhodina* PSU-M35 and PSU-M114, were studied for secondary metabolite production because they showed antibacterial activity against *S. aureus*, both standard ATCC 25922 (SA) and methicillin-resistant (MRSA) strains. (*R*)-(-)-mellein (**1**), (3*R*,4*R*)-4-hydroxymellein (**18**), (3*R*,4*S*)-4-hydroxymellein (**20**) and (3*R*)-5-hydroxymellein (**19**) were recognized by spectroscopic and physic properties. Compounds **1**, **18**, **19** and **20** showed good activity against both strains; however, the most active compound isolated was (3*S*)-lasiodiplodin [45].

*Tubercularia* sp. TF5 was isolated from the bark of *Taxus mairei* collected in Fujian Province, southeast China. Taxol, the well-known anticancer compound, was produced by this strain. Thus, it was studied for the production of other bioactive metabolites. The chromatographic purification of the culture filtrate extract yielded 5-carboxymellein (**12**) which was identified by spectroscopic data [46].

*Neofusicoccum parvum* is the one of the most virulent Botryosphaeriaceae species causing heavy grapevine trunk diseases. (3*R*,4*R*)-4-hydroxymellein (**18**), (3*R*,4*S*)-4-hydroxymellein (**20**), isosclerone and tyrosol were produced by this fungus and identified by spectroscopic methods. All the compounds were tested to evaluate their phytotoxic activities on tomato cuttings at different concentrations, and they showed toxicity ranging from slight to drastic leaf wilting, and compound **18** showed the highest phytotoxicity [47]. *Diplodia africana,* the causal agent of branch dieback on *Juniperus phoenicea,* belonging to the same fungal family of the Botryosphaeriaceae, produced phototoxic metabolites [48]. Two novel phytotoxic dihydrofuropyran-2-ones, named afritoxinones A and B, were isolated, together with known compounds, from the culture filtrates of *D. africana*. Besides afritoxinones A and B, the three well-known melleins (*R*)-(-)-mellein (**1**), (3*R*,4*R*)-4-hydroxymellein (**18**) and (3*R*,4*S*)-4-hydroxymellein (**20**) were isolated [48].

Endophytic fungi living in the intracellular spaces of plants are able to synthesize novel bioactive secondary metabolites. Indeed, the endophytic fungal strain BCRC 33717, obtained from the bark of the medicinal plant *Cinnamomum* sp., produced a plethora of secondary metabolites, including four melleins, which were recognized as the 5-formyl- (**11**), 5-carboxy- (**12**) and 5-hydroxy- (**19**) mellein, and the novel (3*S*)-5-hydroxy-8-*O*-methylmellein (**24**, Figure 2) [49]. Their structures were determined by NMR and MS methods [49]. *Annulohypoxylon squamulosum* BCRC 34022, obtained from the same medicinal plants, when grown on a long-grain rice fermented culture, synthesized eleven different metabolites, which were extracted with *n*-BuOH-soluble 95% EtOH. Among the secondary bioactive metabolites purified from these extracts, three melleins were characterized by spectroscopic methods as(+)-(*S*)-mellein (**2**), (*S*)-7-hydroxymellein (**25**, Figure 2), and (*S*)-5-hydroxymellein (**26**, Figure 2). [50]. Compounds **25** and **26** were isolated for the first time and are the enantiomers of the previously reported compounds **10** and **19**. When assayed on MCF-7 (human breast adenocarcinoma), NCIH460 (non-small-cell lung cancer) and SF-268 (glioblastoma cells), compound **2** possessed moderate toxicity against MCF-7, NCI-H460 and SF-268 cancer cell lines, while **25** and **26** showed weak to negligible activities against all the three cell lines [50].

To keep talking about endophytic fungi, *Epicoccum nigrum*, an ascomycete fungus distributed worldwide, colonizes different types of soils and host plants, and was used as a biocontrol agent for plant pathogens. *E. nigrum* wild type P16 produced secondary metabolites, including (*R*)-(-)-mellein (**1**) [51]. Three *E. nigrum* agro-transformants, namely, P16–17, P16–47set and P16–91, were studied in order identify the genes related to the synthesis of a new natural compound produced by *E. nigrum* P16. The comparison of the extracts of the wild type and the transformants by GC-MS, revealed that the mutants were capable of producing (*R*)-5-hydroxymellein (**19**) as well [51].

*Biscogniauxia nummularia* (Bull.), a seemingly endophytic fungus isolated from the plum yew *Cephalotaxus harringtonia*, produced a new guaiane sesquiterpene together with a previously known terpenoid, xylaranol B, and two mellein derivatives identified as 5-methylmellein (**4**) and 8-methoxy-5-methylmellein (**22**) [52].

*Seimatosporium* sp. was studied for its ability to synthesize biologically active compounds in a program planned to investigate endophytic fungi for new products for pharmacy and plant protection [53]. (*R*)-(-)-Mellein (**1**), (3*R*,4*R*)-4-hydroxymellein (**18**), (3*R*,4*S*)-4-hydroxymellein (**20**), (3*R*)-5-hydroxymethylmellein (**14**) and (3*R*,4*S*)-4-hydroxy-5-methylmellein (**17**) were isolated from fermentation extracts of *Seimatosporium* sp. and identified by spectroscopic methods [53].

Two novel succinic acid derivatives, xylacinic acids A and B, were isolated, along with (*R*)-8-methoxymellein (**9**), (3*R*)-5-methoxycarbonylmellein (**13**) and (3*R*)-5-hydroxymellein (**19**), from the mangrove-derived fungus *Xylaria cubensis* PSU-MA34 [54]. Al the metabolites isolated were tested for antibacterial activity against *S. aureus* ATCC 25923 and MRSA and for cytotoxicity against KB cells; however, the melleins were inactive [54].

The extracts from eleven fungal strains associated with *Eudistoma vannamei* were assayed against two cell lines asthe colon and melanoma cancer cell lines [55]. *Aspergillus* sp. yielded the most promising extract. Its potato dextrose broth extract was purified by bio-guided chromatographic method, and (*R*)-(-)-mellein (**1**), (3*R*,4*R*)-4-hydroxymellein (**18**) and (3*R*,4*S*)-4-hydroxymellein (**20**) were isolated, along with penicillic acid. All isolated compounds were tested for their cytotoxicity; however, only penicillic acid showed cytotoxic activity (cell growth inhibitions > 95%) [55].

The bioactive metabolites were produced by an endophytic fungus, identified as *Nigrospora* sp. by rDNA sequence analysis, and four of them were identified by comparison of the ^1^H-NMR and ^13^C-NMR spectroscopic data with those previously reported. Among them, (*R*)-(-)-mellein (**1**) was identified and showed only weak antifungal activity [56]. In another investigation the crude extracts from cultures of *Pezicula livida* were studied for larvicidal activity. The bio-guided chromatographic purification of the extract also afforded (*R*)-(-)-mellein (**1**), and its larvicidal activity was reported the first time with an LC_50_ value of 1.4 ppm against *Aedes aegypti* [57].

An interesting study was conducted by Yun and co-authors (2013), who added NaBr to the fermentation of the fungus *Aspergillus ochraceus* [58]. This resulted in the production of a new brominated mellein analogue; namely, (3*R*)-5-bromomellein (**27**, Figure 2). Furthermore, three known compounds, (*R*)-(-)-mellein (**1**), clavatol and circumdatin A, were also recognized in the same culture. The structure of **27** was assigned through spectroscopic data analyses. All the isolated compounds showed mild radical scavenging activity against 1,1-diphenyl-2-picrylhydrazyl (DPPH) [58].

The organic extract from culture broths of *Arthrinium* state of *Apiospora montagnei* afforded the main metabolites, which were identified as (*R*)-(-)-mellein (**1**) and (3*R*,4*R*)-4-hydroxymellein (**18**) according to their spectroscopic and physical data. In addition, their activity against *Schistosoma mansoni* (adult worms) was tested. Despite the structural similarity, **1** caused the death of 100% of parasites (both male and female) at 200 μg/mL, whereas compound **18** caused the death of 50% of adult worms at 12.5 μg/mL and 100% at 50 μg/mL [59].

*Xylaria* sp. PSU-G12, isolated from *Garcinia hombroniana*, when grown in liquid culture, yielded a corresponding organic extract displaying antioxidant activity [60]. A plethora of secondary metabolites were isolated and characterized by spectroscopic methods, and among them a novel mellein analogue was isolated and characterized as (3*R*,4*R*)-5-carbomethoxy-4-hydroxymellein, and then named xylarellein (**28**, Figure 2). Three known melleins were also identified as (3*R*)-5-methylmellein (**4**), (3*R*)-5-carboxylmellein (**12**) and (3*R*)-5-methoxycarbonilmellein (**13**). All the isolated compounds were evaluated for antioxidant activity in DPPH assays, but none of them exhibited antioxidant activity, highlighting the possibility that the antioxidant activity of the crude extract may involve a synergistic effect [60].

Two formerly undescribed polyketide metabolites were obtained from the cultures of an endophytic fungus isolated from *Meliotus dentatus*. The two compounds appeared to be, based on spectroscopic data, a new mellein named *cis*-4-acetoxyoxymellein (**29**, Figure 2) and one of its derivatives [61]. The two compounds were tested in an agar diffusion assay for their antifungal, antibacterial and algicidal activities against *Botrytis cinerea*, *Septoria tritici*, *Phytophthora infestans*, *Microbotryum violaceum*, *Escherichia coli*, *Bacillus megaterium* and *Chlorella fusca*. Both metabolites displayed strong antibacterial activity, especially towards *Escherichia coli* and *Bacillus megaterium*. In addition, both displayed algicidal activity against *C. fusca* and good antifungal activity against *M. violaceum*, *B. cinerea* and *S. tritici* [61].

The endophyte *Xylaria* sp., isolated from a surface-sterilized Concord grape leaf (*Vitis labrusca*), grown in liquid culture, produced seven compounds, among which two mellein derivatives were isolated and identified by spectroscopic data as (3*R*)-5-methoxycarbonylmellein (**13**) and (3*R*)-5-carboxylmellein (**12**). However, no biological activities were tested for these two metabolites [62].

(*R*)-(-)-mellein (**1**) was also produced by *Lasiodiplodia* sp. ME4-2, an endophytic fungus obtained from the floral sections of *Viscum coloratum* [63], and from *Pezicula* sp., an endophyte obtained from twigs of *Forsythia viridissima*, Zhejiang Province, Southeast China [64]. In this latter study, (*R*)-(-)-mellein (**1**) showed growth inhibition against nine plant pathogenic fungi, especially *Botrytis cinerea* and *Fulvia fulva* with EC_50_ values below 50 μg/mL [64].

From the solid culture of the endophytic fungus *Xylaria* sp. SNB-GTC2501, which was obtained from the leaves of *Bisboecklera microcephala*, the two already known mellein derivatives **4** and **12** were obtained, whose antimicrobial potential was assessed against human pathogens (i.e., *Staphylococcus aureus*, *Trichophyton rubrum* and *Candida albicans*). However, their minimal inhibitory concentrations were more than 128 μg/mL. In addition, **4** and **12** were not cytotoxic towards MRC5 cells (IC_50_ >100 μM) [65].

(3*R*)-5-methylmellein (**4**), (3*R*)-5-formylmellein (**11**) and (3*R*)-5-carboxymellein (**12**), were also synthesized, together with two undescribed dihydronaphthalenones and the known 2,6-dihydroxy-4-methylacetophenone, by the endophytic fungus *Nodulisporium* sp., isolated from *Antidesma ghaesembilla*. No biological activities were reported for the mellein derivatives **4**, **11** and **12 [66]**.

The endophytic fungus *Botryosphaeria* sp. KcF6 derived from the mangrove plant *Kandelia candel* produced (3*R*)-8-methoxymellein (**9**) and (3*R*,4*S*)-4-hydroxylmellein (**20**), which were identified by spectroscopic data [67]. The isolated compounds were evaluated for their cytotoxic and anti-inflammatory (COX-2) activities, but **9** and **20** were inactive [67].

The previously undescribed (3*R*)-5-ethoxycarbonylmellein (**30**, Figure 2) was isolated from the fungus *Marasmiellus ramealis* isolated in China together with the known **13** and other nine compounds. The structure of the new compound was elucidated by spectroscopic methods, but no activity was reported [68].

The ability of *N. parvum* to produce phytotoxins was further investigated by Abou-Mansour et al. [69]. (*R*)-(-)-mellein (**1**), (3*R*,4*R*)-4-hydroxymellein (**18**) and (3*R*,4*S*)-4-hydroxymellein (**20**), already reported as phytotoxic metabolites produced by another strain of *N. parvum* [47], were isolated together with 3-hydroxymellein and other compounds belong to different chemical families. 3-Hydroxymellein (**31**, Figure 3) was previously isolated from *Aspergillus oniki* 1784 [19], but this study provided its first ^1^H and ^13^C NMR data. Its 3*R* AC was assigned by comparing its CD spectrum with that of **1**.

The phytotoxicity of the main isolated compounds were assessed 48 h after-inoculation at concentrations of 100 and 200 μg/mL, on leaf discs of *Vitis vinifera* cv. Chardonnay. All the compounds tested induced necrosis on host plant leaves, and among the four melleins, (3*R*)-3-hydroxymellein (**31**) was the most active at the lower concentration [69].

Six species of *Lasiodiplodia* isolated in Brazil and causing Botryosphaeria dieback of grapevines were studied to evaluate their production of phytotoxic secondary metabolites. Some mellein derivatives were produced, as ascertained by LC/MS. In particular, *Lasiodiplodia brasiliense* synthesized (3*R*,4*S*)-4-hydroxymellein (**20**), and *Lasiodiplodia euphorbicola* produced (*R*)-(-)-mellein (**1**), (3*R*,4*R*)- and (3*R*,4*S*)- 4-hydroxymellein (**18** and **20**) [70].

The production of **1** by the endophyte *Lasiodiplodia theobromae* was confirmed in a study on the metabolomics-guided isolation of anti-trypanosomal metabolites. However, **1** was inactive when tested against *Trypanosoma brucei brucei* [71]. Compound **1** was also isolated together tyrosol and a new isochromanone, named fraxitoxin, from liquid cultures of *Diplodia fraxini*, a pathogen involved in the etiology of canker and dieback disease of *Fraxinus* spp. in Europe [72].

*Paraconiothyrium sporulosum* YK-03, a marine-derived fungus, also produced **1** together with seven mellein derivatives, including the previously undescribed (3*S*,4*S*)-4,5-dihydroxymellein (**32**, Figure 3) and (*R*)-(-)-mellein-8-*O*-β-D-glucopyranoside (**33**, Figure 3). Their structures and ACs were determined by comprehensive spectroscopic and computational electronic circular dichroism (ECD). Furthermore, their monosaccharide composition was determined by HPLC coupled with optical rotation detector. The known compounds were identified by spectroscopic methods as (3*R*)-7-hydroxymellein (**10**), (3*R*)-5-hydroxymellein (**19**), (3*R*,4*R*)-4-hydroxymellein (**18**), (3*R*,4*S*)-4-hydroxymellein (**20**) and (3*R*,4*S*)-4,5-dihydroxymellein (**34**, Figure 3) [73]. Compound **34** was previously isolated from the marine fungus *Phomopsis* sp. (number ZH-111) [73]. All the compounds were assessed for their cytotoxic activities against the human cancer cell lines A549 and MCF-7. However, none of them showed meaningful cytotoxicity against these two cell lines [74].

The endophytic fungus *Lachnum palmae*, obtained from *Przewalskia tangutica,* was exposed to a histone deacetylase inhibitor SAHA and produced seven previously undescribed halogenated dihydroisocoumarins, named palmaerones A-G (**35**-**41**, Figure 3), together with eleven known dihydroisocoumarins. They were identified by spectroscopic and optical methods as **1**, **3**, **18**, **19**, **20**, (3*R*)-5-bromo-6-hydroxy-8-methoxymellein (**35**), (*3R*)-7-bromo-6-hydroxy-8-methoxymellein (**36**), (3*R*)-7-bromo-6,8-dimethoxymellein (**37**), (3*R*)-7-bromo-6-hydroxy-mellein (**38**), (3*R*)-5-bromo-6,7-dihydroxy-8-methoxy-mellein (**39**), (3*R*)-5-chloro-6-hydroxy-8-methoxy-mellein (**40**), (3*R*)-7-chloro-6-hydroxy-8-methoxy-mellein (**41**), (3*R*)-5-cholro-6-hydroxymellein (**42**, Figure 3), (3*R*,4*R*)-5-cholro-4,6-dihydroxymellein (**43**, Figure 3), (3*R*)-6-hydroxymellein (**44**, Figure 3), (3*R*)-5-bromo-6-hydroxy-8-methoxy-mellein, named palmaerone A (**45**, Figure 3), (3*R*)-7-bromo-6-hydroxy-8-methoxy-mellein, named palmaerone B (**46**, Figure 3) and (3*R*)-7-bromo-6-hydroxy-mellein, named palmaerone D (**47**, Figure 3) [75]. The three melleins **45**-**47** were previously isolated from the same fungus together with the other mellein derivative, namely, (3*R*)-7-bromo-6,8-dimethoxy-mellein, which was named palmerin C (**48**, Figure 3) [76]. Compounds **35**-**41** showed antimicrobial activities against the strains of *Candida neoformans*, *Penicillium* sp., *Candida albicans*, *Bacilus subtilis* and *S. aureus*, and compound **39** showed potential antimicrobial activity against all the test strains with the MIC value in the range of 10–55 μg/mL. Metabolites **35** and **39** exhibited mild inhibitory effects on nitric oxide (NO) production in lipopolysaccharide (LPS)-induced RAW 264.7 cells, with the IC_50_ values of 26.3 and 38.7 μM, respectively and no evident toxicities were observed at 50 μM. Compound **39** showed weak cytotoxicity against HepG2 with the IC_50_ value of 42.8 mM [76].

An interesting study was carried out on 579 fungal culture extracts to identify the bioactive compounds capable of inhibiting histone deacetylase activity of Sirtuin A (SirA), produced by the fungus *Aspergillus nidulans* [77]. Sirtuin is a family of histones that are implicated in fungal growth and secondary metabolite production. Eight fungal strains, belonging to different families, showed to produced SirA inhibitors, and among them, *Didymobotryum rigidum* JCM 8837 showed to produce 5-methylmellein (**4**). This mellein derivative was found to inhibit SirA activity with IC_50_ of 120 μM. In addition, in another experiment compound **4** was added to *A. nidulans* cultures. This resulted in an increased secondary metabolite production. In conclusion, the results highlighted that 5-methylmellein (**4**) can modulate fungal secondary metabolism and is a potential tool for screening novel compounds derived from fungi [77].

Compound **1** was isolated from the culture filtrates of *Diplodia mutila* FF18 *Diplodia seriata* H141a, *Neofusicoccum australe* VP13 and *Neofusicoccum luteum,* as resulted from a recent study on phytotoxic metabolites produced by nine species of Botryosphaeriaceae involved in grapevine dieback in Australia. From *N. luteum,* **18** and **20** were also isolated [78].

*Sardiniella urbana*, a pathogen of European hackberry trees in Italy, was investigated for its ability to produce secondary metabolites. It produced **1**, **18** and **20,** which were identified by spectroscopic methods. These compounds were assayed for their phytotoxic, antifungal and zootoxic activities, and among them, only (*R*)-(-)-mellein was found to be active. In particular, **1** displayed from significant to weak activity towards all plant pathogens tested at 0.2 mg/plug. *Athelia rolfsii*, *Botrytis cinerea* and *Sclerotinia sclerotiorum* were the most sensitive species. On the contrary, *Alternaria brassicicola*, *Fusarium graminearum* and *Phytophthora cambivora* were less sensitive. In the *Artemia salina* bioassay, **1** caused 100% larval mortality at 200 μg/mL. The LC_50_ value was 102 μg/mL after 36 h of exposure to the metabolite [79].

*Aspergillus flocculus*, an endophyte isolated from the stem of the medicinal plant *Markhamia platycalyx,* was investigated for its ability to synthesize bioactive anticancer and anti-trypanosome secondary metabolites. From the fermentation culture of the fungus were isolated several metabolites belonging to different classes of natural compounds. Among them were isolated some mellein derivatives identified as **1, 18**, **19**, **20**, **31**, **34** and botryoisocoumarin A (**49**, Figure 3). Compounds **18**, **34**, **49** and **1** inhibited the growth of chronic myelogenous leukemia cell line K562 at 30 μM. Compound **31** exhibited an inhibition of 56% to the sleeping-sickness-causing parasite *Trypanosoma brucei brucei* [80]. Compound **49**, characterized as (3*R*)-3-methoxymellein, was also previously obtained from the fermentation culture of *Botryosphaeria* sp. F00741, isolated from the plant epidermis of *Avicennia marina* [81].

Compound **1** was also recently isolated from the endophytic fungus *Colletotrichum gloeosporioides* GT-7, obtained from *Uncaria rhynchophylla* [82].

*Xylaria* sp. SWUF09-62, a Basidiomycota fungus belonging to the Xylariaceae family, was investigated to explore its ability to produce natural products with anti-inflammation and anti-proliferation activities [83]. This research led to the isolation of several melleins derivatives which were identified by spectroscopic methods as (3*S*)-7-methoxymellein (**50**, Figure 3) and (3*S*)-5,7-dihydroxymellein (**51**, Figure 3), and their ACs were determined by ECD experiments. (3*S*)-8-methoxymellein (**52**, Figure 3), previously synthesized by Kerti et al. [84], was isolated for the first time as a natural compound. In addition, (*S*)-(+)-mellein (**2**), (3*R*)-5-methoxycarbonylmellein (**13**), (3*R*,4*R*)-4-hydroxymellein (**18**), (3*R*,4*S*)-4-hydroxymellein (**20**) and (3*S*)-7-hydroxymellein (**25**), were also isolated. Anti-inflammatory activity screening was carried out by measuring the reduction of NO production in LPS-induced RAW264.7 macrophage cells, and the mellein derivatives showed different degrees of activity. Compound **51** exhibited anti-inflammatory properties by reducing nitric oxide production in LPS-stimulated RAW264.7 cells, indicating possible chemo-preventative and chemo-therapeutic properties [83].

The study conducted by Inose et al. [85] demonstrated the potential of density functional theory (DFT)-based calculations and ECD spectral calculations for structural elucidation of natural compounds. The extract of *Periconia macrospinosa* KT3863 was studied for secondary metabolite production and two new chlorinated melleins, (3*R*)-5-chloro-4-hydroxy-6-methoxymellein and (3*R*)-7-chloro-6-methoxy-8-methoxymellein (**53** and **54**, Figure 3), were isolated. Furthermore, the authors reported for the first time the complete characterization of the physical properties of the previously isolated (3*R*)-5-chloro-6-methoxymellein (**55**, Figure 3) [86]. The results of (DFT)-based calculations were used to estimate the values of the ^13^C chemical shifts and the spin coupling constants and compare them with experimental data collected by HMBC experiment. The calculations allowed them to determine the relative configurations of **53**. In addition, the ACs of **53–55** were established by comparing the experimental ECD spectra with those obtained by time-dependent DFT calculations. The data showed that **53** afforded an ECD spectrum that was almost the mirror image of that of **54**. Finally, the authors studied the antifungal activities of **53–55** with *Cochliobolus miyabeanus* as the model organism; unfortunately no significant inhibition was observed [85].

### 2.2. Melleins from Plants

The first mellein derivative from a plant was (3*R*)-6-methoxymellein (**3**) isolated from bitter carrots (*Daucus carota*) in 1960 [87]. Successively, compound **3** was isolated in higher yields from carrot root tissue (*D. carota*) inoculated with *Ceratocystis cimbriata*, *Ceratocystis ulmi*, *Helminthosporum carbonum* or *Fusarium oxysporum* [88]. After this investigation, it was hypothesized that the production of **3** could resulted from an alteration of the normal metabolism of the plant induced by the presence of fungi together with environmental condition [88]. This proposal was elaborated in a 1963 review, and 6-methoxymellein (**3**) was classified as a phytoalexin [89]. Thus, 6-methoxymellein (**3**) plays an important role on the active defenses of whole cold-stored carrots and this property was further investigated [90]. The ethanolic extract of cold-stored carrots slices was purified by TLC and the compounds identified by spectroscopic methods. The isolated compounds were assessed against spore suspensions of *Botrytis cinerea.* In the spore germination bioassay, the most active inhibitor effect was induced by 6-methoxymellein (**3**) [90].

Recently, ten different secondary metabolites were isolated from the methanol extract of the twigs and leaves of *Garcinia bancana*, and their structures were elucidated by spectroscopic methods. Among them (*R*)-(-)-mellein (**1**) was identified [91]. In the same year, compound **1** was isolated from the organic extract the stems of *Ficus formosana* (Moraceae) [92]. Compound **1** was also isolated from the extract of *Enicosanthum membranifolium* together with clerodermic acid and salicifoline, whose identities were confirmed by using X-ray diffractometric analysis [93]. The extract of the wood of *Millettia leucantha* proved to contain (*R*)-(-)-mellein (**1**) and other secondary metabolites, and the structures of these compounds were assigned by the analysis of their spectroscopic data [94].

Chemical constituents of the whole herb extract of *Rhodiola kirilowii* Maxim were purified and identified using 1D and 2D NMR methods. Eleven compounds were obtained, and one of them was identified as (*R*)-(-)-mellein (**1**) and isolated for the first time from *Rhodiola* genus [95].

Compound **1** was also isolated from the roots of *Antidesma acidum*, as identified by spectroscopic methods [96]. (*R*)-(-)-mellein (**1**) was also isolated from the roots of another plant *Microcos tomentosa* [97]. In the same year, from the extract of leaves and stems of *Stevia lucida* Lagasca, different compounds were isolated, and among them, **1** was identified on the basis of its spectroscopic properties. This was the first report of **1**, and in general of an isocoumarin, in *Stevia* genus [98].

6-Methoxymellein (**3**), three undescribed and two known xanthones and three biflavanoids were isolated from the methanolic extract of the twigs of *Garcinia xanthochymus*. Their structures were identified by spectroscopic data; unfortunately the amount of **3** was too low to conduct any bioassay [99].

Masatoshi and co-authors studied the attractiveness of several wood odors to beetles. The beetles were highly attracted to all wood odors of *Castanea crenata, Magnolia obovata, Paulownia tomentosa, Prunus jamasakura* and *Zelkova serrata*. The *Z. serrata* supercritical CO_2_ extract was the most attractive extract and was analyzed by GC-MS. The major compound detected in the extract was (*R*)-(-)-mellein (**1**) and proved to attract the beetles [100]. In the same year, a new bianthraquinone, named by morindaquinone, together with another 12 known secondary metabolites, was isolated from the roots of *Morinda coreia*. Among those, **1** was isolated and identified according to its spectroscopic data [101].

### 2.3. Melleins from Insects

The swarming of the carpenter ant, *Camponotus herculeanus*, is influenced by climatic factors, such as season, temperature and time of day. The synchronization of this swarming is controlled by volatile compounds secreted from the mandibular glands of the males [102]. Brand and his colleagues (1973) analyzed, by GC-MS, the major volatiles in the mandibular gland secretions of *C. herculeanus*, *Camponotus ligniperda* and *Camponotus pennsylvanicus*. The analysis showed that the secretions were dominated by two substances, (*R*)-(-)-mellein (**1**) and methyl 6-methylsalicylate, which were further characterized also by NMR data [103]. Another chemical-ecology study on a different species of ant (*Rhytidoponera metallica* workers) was carried out by Brophy and coauthors in 1981. Thirteen volatile constituents have been characterized in an Australian representative of the primitive ant subfamily Ponerinae, called *R. metallica* by GC-MS spectrometry. The major component of the total extracts from the bodies of *R. metallica* workers was (*R*)-(-)-mellein (**1**). However, its glandular origin in the gasters of *R. metallica* is unknown [104].

The production of **1** by ant *Camponotus vagus* was studied in view of its possible chemotaxonomic and functional significance d [105]. (*R*)-(-)-mellein (**1**) was detected as a mandibular gland product of workers of two *Camponotus* species, and it was also detected in both females and males. Furthermore, compound **1** was also isolated from another formicine species, *Polyrhachis doddi*. These results highlighted that (*R*)-(-)-mellein (**1**) is a characteristic compound of the chemical ecology of ants and is clearly a part of an ant’s defensive exudate [105].

More recently, the volatile components of whole-body extracts of four species of neotropical ants in the formicine genus, as *Camponotu kaura*, *Camponotum sexguttatus*, *Camponotu ramulorum* and *Camponotu planatus,* were investigated. Volatile mandibular gland compounds were found only in male extracts in three of the species. The results were different within the species; in particular, (*R*)-(-)-mellein (**1**) was found only in traces in the *C. ramulorum* species. In addition, the significance of the mandibular gland secretion for formicid systematics was also discussed [106]. In another study, six compounds were identified in the heads of *Camponotus irritibilis*; among them (*R*)-(-)-mellein (**1**) and 4-hydroxymellein were isolated and identified. Unfortunately, the authors of the study did not assign the absolute configuration to the latter compound. The possibility of semiochemical thrift for these mandibular gland compounds was reviewed and compared with existing data on mandibular gland compounds of other ants of this group [107].

Male wing gland secretion and volatiles emanated from *Aphomia sociella* (Bee moth) were studied [108]. Two-dimensional gas chromatography-mass spectrometry (GCxGC-TOF-MS), gas chromatography-infrared spectroscopy (GC-FTIR), enantioselective gas chromatography (Es-GC), electroantennography (EAG), gas chromatography with electroantennographic detection (GC-EAD) and NMR were used. The GC-EAD analysis of the male wing gland secretion showed seven different compounds, and among them, (*R*)-(-)-mellein (**1**) was identified. Compound **1** and the nona-2,6-dien-4-olide were the most abundant compounds. One or more of these compounds might constitute sex pheromones [108].

Social insects have developed strong antimicrobial defenses against infection of pathogens and parasites. Indeed, antimicrobial compounds have been identified in *Reticulitermes speratus* (Kolbe) organic extracts. Mitaka and his colleagues (2019) identified (*R*)-(-)-mellein (**1**) using GC-MS analysis. Antifungal assays showed that compound **1** has an inhibitory effect on the growth of *Metarhizium anisopliae* and *Beauveria bassiana*. These results suggest that *R. speratus* use (*R*)-(-)-mellein (**1**) to fight the pathogenic fungi; unfortunately the termite-egg-mimicking fungus has resistance against **1** [109].

### 2.4. Melleins from Bacteria

Only one article on (*R*)-(-)-mellein (**1**) produced by bacteria is available. Volatile compounds released by 50 bacterial strains have been collected, and the obtained headspace extracts were analyzed by GC-MS, which is a fundamental tool for the discovery of natural compounds that might be missed by using traditional techniques [110]. Furthermore, *Saccharopolyspora erythraea* was found to produce compound **1** for the first time. Aside from (*R*)-(-)-mellein (**1**), other insect pheromones such as methyl 6-methylsalicylate, methyl 6-ethylsalicylate, pyrrole-2-carboxylate, conophthorin and chalcogran were produced by bacteria strains. Considering the symbiotic relationships between actinomycetes and insects, further investigations should be performed on the origins of these compounds in these species [110].

## 3. Biosynthetic Pathways and Gene Involved in Mellein Production

Isocoumarins, 3,4-dihydroisocoumarins and melleins belong to the class of secondary metabolites named polyketides. Considering their activities and their biological roles, this class of natural compounds is one of the major secondary metabolite classes. In general, they occur in fungi, plants, bacteria and marine organisms. Isocoumarines, 3,4-dihydroisocoumarins and melleins show a common biosynthetic origin: they are related to the fatty acid biosynthesis, which reactions are catalyzed by enzymes named polyketides synthase (PKS) [111].

**Figure 4:**
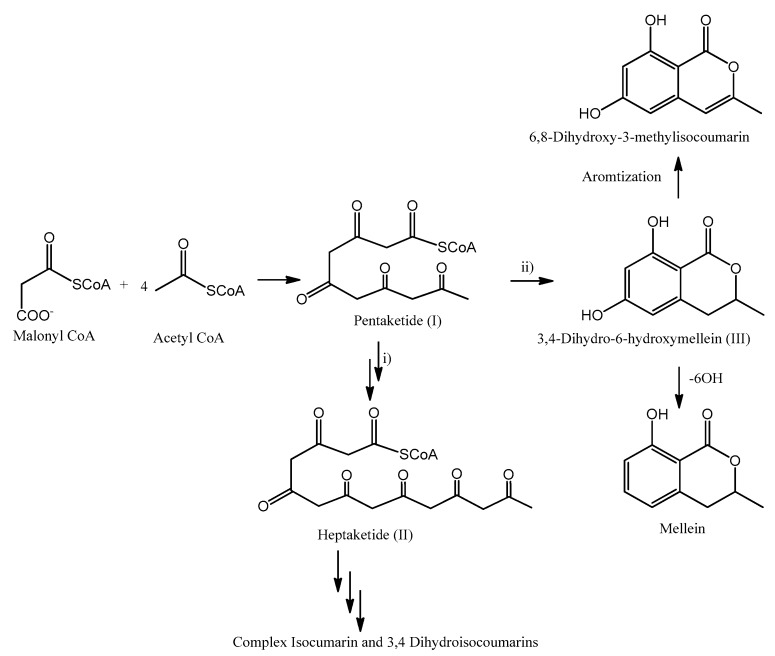
Possible reactions involved with isocoumarines, 3,4-dihydroisocoumarins and melleins biosyntheses.

The possible sequence of reactions involved in their biosynthesis is outlined in Figure 4. Starting form malonyl-CoA and successive Claisen condensation with 4 acetyl-CoA moieties, pentaketide (I) is generated. This reaction initially produces a β−chetoester, and then the ketonic group is reduced after each stage of condensation and before the subsequent phase of chain elongation [111]. Pentaketide (I) might be involved in different reactions: (i) Further chain elongation, spawning the heptaketide (II). Post-PKS modification of II may result in a variety of more complex isocoumarines or 3,4-dihydroisocoumarins. (ii) Cyclization reaction, which produces the typical six-membered lactone ring synthetizing 6-hydroxymellein (III). Further modification of III my include aromatization, generating 6,8-dihydroxy-3-methylisocoumarin, or 6-OH dehydration, forming mellein [111,112].

From detailed investigations of genes, amino acid sequences and mechanistic analogies of the enzymes, were possible to identified three general types of PKS: (i) type I, which are very big multifunctional proteins with a single domain. Furthermore, they can also be divided into iterative and non-iterative enzymes; (ii) type II, composed by complex, single, monofunctional proteins; (iii) type III, which differ from the other two by being homodimeric proteins that use a single active site to perform the series of decarboxylation, condensation, cyclization and aromatization reactions. PKS type III are found in plants, bacteria and fungi, PKS type I are typical of bacteria and fungi, while type II are limited to bacteria. Aromatic polyketides, such as melleins, are typical products of PKS type II or type III, although there are some examples of PKS type I capable of producing aromatic rings [111]. As reviewed in the previous section melleins are mainly produced by pathogenic fungi. Melleins, and more in general polyketides, play a wide range of roles: host-pathogen interaction, facilitations of the host colonization, phytotoxicity [113]. Almost all fungal PKS currently known are type I systems, while some fungi also possess type III PKS [114]. Nevertheless, the fungal type I PKS differs from bacterial type I in being iterative [111,114]. Fungal PKS have different domains: (i) no reductive PKS (nrPKS) with no reductive steps during chain construction, (ii) partially reducing PKS (prPKS), that usually catalyzes only one reduction during chain extension and (iii) highly reducing PKS (hrPKS) where the level of reduction is varied and clearly subject to a high level of genes expression control [114].

Fungi usually have 20–50 secondary metabolites genes and their production is highly regulated often in response to specific biotic factors and environmental perturbations. Modern genomic and transcriptomic tools can be used, for pathogenic fungi, to probe the expression of secondary metabolites gene clusters at various stages of infection [115,116]. Unfortunately, the absence of whole genome sequences slows down the identification of these target genes.

Focusing our attention on genes sequences and characterized PKS enzymes involved in melleins production in fungi, very little it is available in literature so far.

*Saccharopolyspora erythraea*, an actinomycete that produces a polyketide with antibiotic activity named erythromycin A was studied [117]. The modular PKS appointed for the biosynthesis of erythromycin A was studied as model for polyketide synthesis. The genome of *S. erythraea* revealing a dozen of PKS genes. One of the uncharacterized PKS genes was SACE5532, which encodes a single-module PKS that have sequence homology with several fungal and bacterial type I prPKSs for aromatic polyketide biosynthesis. The product of SACE5532 was identified as (*R*)-(-)-mellein (**1**), and the different domains of this prPKS were studied and characterized. The experimental results confirmed the polyketide origin of **1** and might ease the identification of the biosynthetic genes for other dihydroisocoumarins [117].

More recently, the sequence of fungal PKs involved in (*R*)-(-)-mellein (**1**) synthesis in wheat pathogenic fungus *Parastagonospora nodorum* was reported and the gene, involved in the production of **1** by the wheat pathogen *P. nodorum*, was studied [118]. The results showed that SN477 was the most highly expressed PKs gene *in planta*, and analysis of the DNA sequence indicated that it codes for typical prPKS and was similar with an identical domain architecture to the prPKS ATX from *Aspergillus terreus*, which synthesizes 6-MSA. These results were confirmed by heterologous expression of SN477 in yeast. The gene knock-out SN477 resulted in a *P. nodorum* mutant that was not capable of producing (*R*)-mellein as shown by HPLC metabolic profiling. Thus, SN477 is the first fungal prPKS producing a PKs compound except 6-MSA. However, its biosynthesis was highly parallel to that of 6-MSA but needed additional chain elongation and keto reduction steps [118].

## 4. Conclusion and Perspectives

Table 1 summarizes all the isolated melleins and their biological activities.

**1** is the only mellein produced by fungi, plants, insects and bacteria, while 3 is produced by fungi and plants. All the other mellein derivatives (2, 4–55) are produced by fungi. Thus, fungi are the best natural source of this family of natural products, and it is interesting to note that fungi belonging to species even different from a phylogenetic point of view can produce them in vitro and under different cultural conditions.

Melleins have different biological activities and could have a fundamental role in the chemical ecology of the producer microorganisms. The chemical ecology can be defined as the science that studies the chemical mediation that permits living organisms to communicate among themselves and within their environment [119]. Among the different biological activities shown by **1**–**55**, the phytotoxic and the antimicrobial ones are the most observed, suggesting two main rules played by these compounds. The phytotoxic melleins can act as pathogenicity (i.e., the ability to cause disease) or virulence (i.e., the severity of disease) factors in host–pathogen interactions and in the infection process. The melleins that showed antimicrobial activity could be produced to compete with other organisms, reducing or inhibiting the growth of competitors.

Until now it has been very difficult to demonstrate these hypotheses, especially in the case of fungi, for which the first step is to prove their ability to produce melleins *in vivo*. However, recent metabolomics methods, based on the use of multivariate statistical analysis and powerful analytical techniques (LC-NMR, LC-MS/MS, GC-MS/MS), could be very helpful through offering the opportunity to detect active compounds in complex mixtures if present in very low amounts [120]. Thus, these studies could be useful to disclose all the ecological, biological and biochemical roles played by mellein derivatives.

Furthermore, considering that the AC is an important factor to impart biological activity [121,122], some considerations can be made on this point. Most of the melleins possess an *R* configuration at C-3, but some fungi, belonging to *Annulohypoxylon, Aspergillus, Biscogniauxia, Cercospora, Fusarium, Paraconiothyrium, Phomopsis* and *Xylaria* species*,* are able to produce compounds with the *S* configuration at the same carbon as well (namely, compounds **2**, **7**, **8**, **24–26**, **30**, **50–52**). However, these compounds have no phytotoxicity or antimicrobial activities, only cytotoxic (**2**), weak cytotoxic (**25** and **26**) and (**51**) anti-inflammatory proprieties. Thus, it seems that the *R* configuration at C-3 and the different functionalization of the mellein skeleton are important factors with which to modulate and discriminate the biological activities of these compounds.

Finally, further investigations are needed to completely clarify the genes involved in melleins biosynthesis. Indeed, comparative genomics and transcriptomics are important tools that could assist in the identification of the gene clusters involved in secondary metabolites biosynthesis. Comparative genomics might also be used for identifying target gene clusters of a group of secondary metabolites containing structural similarities [123,124]. The recent availability of next-generation RNA-Seq technologies has revolutionized transcriptomic profiling. Indeed, genome sampling and re-sequencing has become a routine, nowadays. However, as suggested by the Chooi and Solomon [125], to obtain further insights into the bio-ecological functions of these secondary metabolites’ gene clusters, the encoded secondary metabolites must first be identified and chemically characterized.

## Figures and Tables

**Table 1:** Naturally-occurring melleins, their natural sources and their biological activities.

Mellein	Natural Source	Biological Activity	References
(*R*)-(-)-mellein (**1**, Figure 1)	Fungi:		
	*Apiospora montagnei*		[40]
		larvicidal	[59]
	*Aspergillus* *flocculus*		[55]
		citotoxicity	[80]
	*Aspergillus melleus*		[3]
	*Aspergillus* *ochraceus*		[21,22,58]
		HCV protease inhibitor	[38]
	*Aspergillus oniki* 1784		[19]
	*Botryosphaeria mamane* PSU-M76		[42]
	*Botryosphearia obtusa*	phytotoxicity	[29,44]
	*Bothryosphaeria rhodina*	antibacterical	[45]
	*Colletotrichum gloeosporioides*		[82]
	*Diplodia africana*		[48]
	*Diplodia fraxini*		[72]
	*Diplodia mutila*		[78]
	*Diplodia seriata*		[78]
	*Epicoccum nigrum*		[51]
	*Eudistoma vannamei*		[55]
	*Hypoxylon deustum*		[26]
	*Hypoxylon fragiforme*		[26]
	*Hypoxylon howeianum*		[26]
	*Hypoxylon haematostroma*		[26]
	*Hypoxylon venusfuissimum*		[26]
	*Lachnum palmae*		[75,76]
	*Lasiodiplodia euphorbicola*		[70]
	*Lasiodiplodia theobromae*		[20,71]
	*Lasiodiplodia* sp.		[63]
	*Microsphaeropsis* sp.	antifungal	[37]
	*Nectria fuckeliana*		[29]
	*Neofusicoccum australe*		[78]
	*Neofusicoccum luteum*		[78]
	*Neofusicoccum parvum*		[69]
	*Nigrospora* sp.		[56]
	*Pezicula livida*	larvicidal	[57]
	*Pezicula* sp.	fungicidal, herbicidal, algicidal	[36,64]
	*Phoma tracheiphila*	phytotoxic	[35]
	*Rhytistimataceae* CBS 120379		[43]
	*Sardiniella urbana*	antifungal	[79]
	*Seimatosporium* sp.		[53]
	*Septoria nodorum*		[24,33]
	*Sphaeropsis sapinea*	phytotoxic, antifungal	[41]
	*Stagonospora apocynin*		[32]
	Plants:		
	*Antidesma acidum*		[96]
	*Enicosanthum membranifolium*		[93]
	*Ficus formosana*		[92]
	*Garcinia bancana*		[91]
	*Microcos tomentosa*		[97]
	*Millettia leucantha*		[94]
	*Rhodiola kirilowii*		[95]
	*Stevia lucida*		[98]
	*Zelkova serrata*		[100]
	Insects:		
	*Aphomia sociella*		[108]
	*Camponotus herculeanus*		[102,103]
	*Camponotus irritibilis*		[107]
	*Camponotus ligniperda*		[103]
	*Camponotus pennsylvanicus*		[103]
	*Camponotus ramulorum*		[106]
	*Camponotus vagus*		[105]
	*Reticulitermes speratus*	antifungal	[109]
	*Rhytidoponera metallica*		[104]
	Bacteria:		
	*Saccharopolyspora erythraea*		[110]
(*S*)-(+)-mellein (**2**, Figure 2)	*Annulohypoxylon squamulosum*	cytotoxicity	[50]
	*Cercospora taiwanensis*		[23]
	*Fusarium larvarum*		[15]
	*Phomopsis oblonga*		[27]
	*Xylaria* sp. SWUF09-62		[83]
(3*R*)-6-methoxymellein (**3**, Figure 2)	Fungi:		
	*Coniothyrium* sp.	antifungal	[37]
	*Lachnum palmae*		
	*Sporormia bipartis*		[16]
	*Sporormia affinis*		[18]
	Plants:		
	*Daucus carota*	phytoalexin	[87,89]
	*Daucus carota*		[88]
	*Garcinia xanthochymus*		[99]
(3*R*)-5-methylmellein (**4**, Figure 2)	*Aspergillus nidulans*	antifungal	[77]
	*Biscogniauxia nummularia*		[52]
	*Fusicoccum amygdali*	conidial germination inhibitor	[17]
	*Hypoxylon* sp.	fungal secondary metabolism	[26]
	*Nodulisporium* sp.		[66]
	*Phomopsis oblonga*	larvicidal	[27]
	*Valsa ceratosperma*	phytotoxic	[28]
	*Xylariaceae* CBS 120381		[43]
	*Xylaria* sp. PSU-G12		[60]
	*Xylaria* sp. SNB-GTC2501		[65]
(3*R*)-7-chloro-6-methoxymellein (**5**, Figure 2)	*Coniothyrium* sp.	antifungal	[37]
	*Sporormia affinis*		[18]
(3*R*)-5,7-dichloro-6-methoxymellein (**6**, Figure 2)	*Sporormia affinis*		[18]
(3*S*,4*S*)-4-hydroxymellein (**7**, Figure 2)	*Aspergillus ochraceus*		[21,22]
	*Cercospora taiwanensis*		[23]
(3*S*,4*S*)-4,6-dihydroxymellein (**8**, Figure 2)	*Cercospora* sp.		[24]
(3*R*)-8-methoxymellein (**9**, Figure 2)	*Apiospora montagnei*		[40]
	*Botryosphaeria* sp. KcF6		[67]
	*Septoria nodorum*		[24]
	*Xylaria cubensis*		[54]
(3*R*)-7-hydroxymellein (**10**, Figure 2)	*Botryosphaeria obtusa*		[44]
	*Paraconiothyrium sporulosum*		[73]
	*Septoria nodorum*		[24]
(3*R*)-5-formylmellein (**11**, Figure 2)	*Biscogniauxia cylindrospora* BCRC 33717		[49]
	*Nodulisporium* sp.		[66]
	*Numularia broomiana*		[26]
	*Numularia discreta*		[26]
	*Xylariaceae* CBS 120381		[43]
(3*R*)-5-carboxymellein (**12**, Figure 2)	*Biscogniauxia cylindrospora* BCRC 33717		[49]
	*Epicoccum nigrum*	phytotoxic	[51]
	*Hypoxylon illitum*		[26]
	*Hypoxylon* *mammatum*		[26]
	*Nodulisporium* sp.		[66]
	*Numularia discreta*		[26]
	*Phomopsis oblonga*	larvicidal	[27]
	*Tubercularia* sp. TF5		[46]
	*Valsa ceratosperma*		[28]
	*Xylaria* sp.		[60]
	*Xylaria* sp. PSU-G12		[62]
	*Xylaria* sp. SNB-GTC2501		[65]
(3*R*)-5-methoxycarbonylmellin (**13**, Figure 2)	*Hypoxylon mammatum*		[26]
	*Marasmiellus ramealis*		[68]
	*Xylariaceae* CBS 120381		[43]
	*Xylaria cubensis*		[54]
	*Xylaria* sp.		[60]
	*Xylaria* sp. PSU-G12		[62]
	*Xylaria* sp. SWUF09-62		[83]
(3*R*)-5-hydroxymethylmellein (**14**, Figure 2)	*Hypoxylon illitum*		[26]
	*Seimatosporium* sp.		[53]
(3*R*)-6-methoxy-5-methylmellein (**15**, Figure 2)	*Hypoxylon atropunctatum*		[26]
	*Valsa ceratosperma*	phytotoxic	[28]
(3*R*,4*R*)-4-hydroxy-5-methylmellein (**16**, Figure 2)	*Valsa ceratosperma*	phytotoxic	[28]
(3*R*,4*S*)-4-hydroxy-5-methylmellein (**17**, Figure 2)	*Valsa ceratosperma*	phytotoxic	[28]
	*Seimatosporium* sp.		[53]
(3*R*,4*R*)-4-hydroxymellein (**18**, Figure 2)	*Apiospora montagnei*	larvicidal	[59]
	*Aspergillus flocculus*		[55]
		citotoxicity	[80]
	*Botryosphaeria mamane* PSU-M76	larvicidal	[42]
	*Botryosphaeria obtusa*	phytotoxic	[30,44]
	*Botryosphaeria rhodina*	antibacterical	[45]
	*Diplodia africana*		[48]
	*Diplodia seriata*		[78]
	*Eudistoma vannamei*		[55]
	*Lasiodiplodia euphorbicola*		[70]
	*Lachnum palmae*		[75,76]
	*Microsphaeropsis* sp.		[37]
	*Neofusicoccum parvum*	phytotocicity	[47,69]
	*Neofusicoccum luteum*		[78]
	*Sardiniella urbana*		[79]
	*Seimatosporium* sp.		[53]
	*Septoria nodorum*		[34]
	*Sphaeropsis sapinea*		[41]
	*Xylaria* sp. SWUF09-62		[83]
(3*R*)-5-hydroxymellein (**19**, Figure 2)	*Aspergillus flocculus*		[80]
	*Botryosphaeria obtusa*	phytotoxic	[30,31]
	*Botryosphaeria rhodina*	antibacterial	[45]
	*Biscogniauxia cylindrospora* BCRC 33717		[49]
	*Lachnum palmae*		[75,76]
	*Paraconiothyrium sporulosum*		[73]
	*Xylaria cubensis*		[54]
(3*R*,4*S*)-4-hydroxymellein (**20**, Figure 2)	*Aspergillus flocculus*		[55,80]
	*Botryosphaerya mamane* PSU-M76		[42]
	*Botryospaheria rhodina*	antibacterial	[45]
	*Botryosphaeria* sp. KcF6		[67]
	*Diplodia africana*		[48]
	*Eudistoma vannamei*		[55]
	*Lachnum palmae*		[75,76]
	*Lasiodiplodia brasiliense*		[70]
	*Lasiodiplodia euphorbicola*		[70]
	*Microsphaeropsis* sp.		[37]
	*Neofusicoccum parvum*	phytotoxicity	[47,69]
	*Neofusicoccum luteum*		[78]
	*Paraconiothyrium sporulosum*		[73]
	*Sardiniella urbana*		[79]
	*Seimatosporium* sp.		[53]
	*Septoria nodorum*		[34]
	*Sphaeropsis sapinea*		[41]
	*Xylaria* sp.		[83]
(3*R*)-7-methoxy-5methylmellein (**21**, Figure 2)	*Cytospora eucalypticola*	middle antibacterial, middle antifungal	[39]
(3*R*)-8-methoxy-5methylmellein (**22**, Figure 2)	*Biscogniauxia nummularia*		[22]
	*Cytospora eucalypticola*	middle antibacterial middle antifungal	[39]
(3*R*)-4,7-dihydroxymellein (**23**, Figure 2)	*Botryosphaeria obtusa*	phytotoxicity	[44]
(3*S*)-5-hydroxy-8-*O*-methylmellein (**24**, Figure 2)	*Biscogniauxia cylindrospora* BCRC 33717		[49]
(3*S*)-7-hydroxymellein (**25**, Figure 2)	*Annulohypoxylon squamulosum*	weak cytotoxicity	[50]
	*Xylaria* sp.		[83]
(3*S*)-5-hydroxymellein (**26**, Figure 2)	*Aspergillus squamulosum*	weak cytotoxicity	[50]
(3*R*)-5-bromomellein (**27**, Figure 2)	*Aspergillus ochraceus*	radical scavenging	[58]
(3*R*,4*R*)-5-carbomethoxy-4-hydroxymellein (**28**, Figure 2)	*Xylaria* sp. PSU-G12		[60]
(3*R*,4*R*)-4-acetoxyoxymellein (**29**, Figure 2)	*Meliotus dentatus*	antibacterial	[61]
(3*R*)-5-ethoxycarbonylmellein (**30**, Figure 2)	*Marasmiellus ramealis*		[68]
(3*R*)-3-hydroxymellein (**31**, Figure 3)	*Aspergillus oniki* 1784	phytotoxicity	[69]
	*Aspergillus flocculus*		[80]
(3*S*,4*S*)-4,5-dihydroxymellein (**32**, Figure 3)	*Paraconiothyrium sporulosum*		[73]
(3*R*)-mellein-8-*O*-β-D-glucopyranoside (**33**, Figure 3)	*Paraconiothyrium sporulosum*		[73]
(3*R*,4*S*)-4,5-dihydroxymellein (**34**, Figure 3)	*Aspergillus flocculus*	cytotoxicity	[80]
	*Phomopsis* sp.		
	*Paraconiothyrium sporulosum*		[73]
(3*R*)-5-bromo-6-hydroxy-8-methoxymellein (**35**, Figure 3)	*Lachnum palmae*	antimicrobial anti-inflammatory	[75,76]
(*3R*)-7-bromo-6-hydroxy-8-methoxymellein (**36**, Figure 3)	*Lachnum palmae*	antimicrobial	[75,76]
(3*R*)-7-bromo-6,8-dimethoxymellein (**37**, Figure 3)	*Lachnum palmae*	antimicrobial	[75,76]
(3*R*)-7-bromo-6-hydroxy-mellein (**38**, Figure 3)	*Lachnum palmae*	antimicrobial	[75,76]
(3*R*)-5-bromo-6,7-dihydroxy-8-methoxy-mellein (**39**, Figure 3)	*Lachnum palmae*	antimicrobial	[75,76]
		anti-inflammatory	[75,76]
		weak cytotoxicity	[75,76]
(3*R*)-5-cholro-6-hydroxy-8-methoxy-mellein (**40**, Figure 3)	*Lachnum palmae*	antimicrobial	[75,76]
(3*R*)-7-cholro-6-hydroxy-8-methoxy-mellein (**41**, Figure 3)	*Lachnum palmae*	antimicrobial	[75,76]
(3*R*)-5-cholro-6-hydroxymellein (**42**, Figure 3)	*Lachnum palmae*		[75,76]
(3*R*,4*R*)-5-cholro-4,6-dihydroxymellein (**43**, Figure 3)	*Lachnum palmae*		[75,76]
(3*R*)-6-hydroxymellein (**44**, Figure 3)	*Lachnum palmae*		[75,76]
(3*R*)-5-bromo-6-hydroxy-8-methoxy-mellein (**45**, Figure 3)	*Lachnum palmae*		[75,76]
(3*R*)-7-bromo-6-hydroxy-8-methoxy-mellein (**46**, Figure 3)	*Lachnum palmae*		[75,76]
(3*R*)-7-bromo-6-hydroxy-mellein (**47**, Figure 3)	*Lachnum palmae*		[75,76]
(3*R*)-7-bromo-6,8-dimethoxy-mellein (**48**, Figure 3)	*Lachnum palmae*		[75,76]
botryoisocoumarin A (**49**, Figure 3)	*Aspergillus flocculus*	cytotoxic	[80]
	*Botryosphaeria* sp. F00741		[81]
(3*S*)-7-methoxymellein (**50**, Figure 3)	*Xylaria* sp. SWUF09-62		[83]
(3*S*)-5,7-dihydroxymellein (**51**, Figure 3)	*Xylaria* sp. SWUF09-62	anti-inflammatory	[83]
(3*S*)-methoxymellein (**52**, Figure 3)	*Xylaria* sp. SWUF09-62		[83]
(3*R*)-5-chloro-4-hydroxy-6-methoxymellein (**53**, Figure 3)	*Periconia macrospinosa*		[85]
(3*R*)-7-chloro-6-methoxy-8-methoxymellein (**54**, Figure 3)	*Periconia macrospinosa*		[85]
(3*R*)-5-chloro-6-methoxymellein (**55**, Figure 3)	*Periconia macrospinosa*		[85]
